# Clavien-Dindo classification for assessment of complications after 1465 unselected otorhinolaryngology and head and neck surgeries in a university hospital: a retrospective observational study

**DOI:** 10.1186/s12893-025-02970-1

**Published:** 2025-05-29

**Authors:** Leonie Glombitza, Jonas Ballmaier, Mussab Kouka, Thomas Bitter, Orlando Guntinas-Lichius

**Affiliations:** Department of Otorhinolaryngology, Jena University Hospital, Friedrich-Schiller-University Jena, Am Klinikum 1, D-07747 Jena, Germany

**Keywords:** Otolaryngology, Otorhinolaryngology, Surgery, Complication, Re-admission, Quality control, Quality management, Bleeding

## Abstract

**Background:**

Assessment of surgical complications is an important part of quality control on departments of otorhinolaryngology and head and neck surgery. Nevertheless, standardized assessment of surgical complications with easy-to-use instruments is not yet clinical routine.

**Methods:**

Data from all 1,465 otorhinolaryngology and head and neck surgeries (male 60.3%; median age: 52 years) performed in 2020 at a department of otorhinolaryngology of a tertiary university hospital were analyzed. The postoperative complications were graded with the Clavien–Dindo classification (CDC).

**Results:**

The most frequent types of surgery were: diagnostic endoscopy (19.4%), ear surgery (14.8%) and oral/pharyngeal surgery (12.1%). Two-hundred seven patients (14.1%) had CDC complications, mostly CDC grade II (6.9%) and CDC grade III (5.1%). Due to multivariate analysis, smoking was an important predictor of postoperative CDC complication (Odds ratio [OR] = 1.526; confidence interval [CI] = 1.037 to 2.244). The risk of re-admission was higher for patients with postoperative complications (OR = 2.859; CI = 2.119 to 3.8591). Compared to a diagnostic endoscopy, the incidence of postoperative complications was highest for esophageal surgery (highest risk: OR = 23.580; CI = 7.619 to 72.978), thyroid surgery (second highest risk: OR = 21.060; CI = 4.838 to 91.665), and salivary gland surgery (OR = 15.330; CI = 5.215 to 45.070).

**Conclusions:**

The CDC grading is a useful tool for grading all kind of otorhinolaryngology and head and neck surgery complications. CDC is well suited for comparing different types of otorhinolaryngology and head and neck surgeries with each other and also patients of different hospitals.

**Supplementary Information:**

The online version contains supplementary material available at 10.1186/s12893-025-02970-1.

## Introduction

A standardized assessment and grading of surgical complications is very important to improve the quality of preoperative, perioperative, and postoperative care. Nevertheless, there is no standard in otorhinolaryngology or head and neck surgery to document complications and to weight their severity. In 1992, Clavien et al. proposed a first simple system for grading of surgical complications based on the type of therapy required to treat the complication [1]. Dindo et al. modified the original proposal and since then the system is named Clavien-Dindo classification (CDC) [2]. Due to its generic approach, the CDC can be applied in any surgical discipline.

PubMed lists more than 2,900 publications for the term “Clavien-Dindo classification” (access date: 06-FEB-2024). The CDC has only rarely been used in otorhinolaryngology or head and neck surgery. It has mainly been used for head and neck cancer surgery [3–6].

In contrast, the classification has rarely been used for other otorhinolaryngology surgeries, only for a perioperative assessment of cochlear implant surgery [7], benign parotid tumor surgery [8], vestibular schwannoma surgery [9], and adenotonsillectomy in children [10].

Hence, a broad spectrum of typical otorhinolaryngology and head and neck surgeries have not yet analyzed with the CDC. Therefore, an unselected and complete series of otorhinolaryngology and head and neck surgeries from the year 2020 from a university hospital, which is also the city hospital, was analyzed. This study included an analysis of patients’ and treatment related factors associated with a higher probability of postoperative complications.

## Materials and methods

### Study design and setting

This retrospective cohort study was carried out at the Department of Otorhinolaryngology, Jena University Hospital. Approval for the study was obtained through the local Institutional Review Board.

### Patients undergoing otorhinolaryngology and head and neck surgeries

The study cohort consisted of all patients admitted to the Department of Otorhinolaryngology for surgery from January to December 2020 (12 months). Demographic and medical variables were measured by chart review. Alcohol consumption was categorized (no/yes). Patients were classified as smokers if they smoked cigarettes or quit smoking ≤ 3 months before surgery. All other patients were classified as non-smokers. Charlson comorbidity index (CCI) was used to measure the general comorbidity of the patients [11]. All patients were classified preoperatively by the anesthesiologists by the American Society of Anesthesiologists (ASA) physical status classification system. ASA is a grading system to determine the health of a person before a surgical procedure that requires anesthesia [12]. It records the physical condition of a patient and is divided into six categories. Group I describes healthy patients, group II is mild general disease, group III is severe general disease without performance limitations, group IV is severe, long-lasting general disease with performance limitations, and group V is a moribund patient not expected to survive without the operation. Group VI includes brain-dead patients.

### Classification of the surgical complication with the Dindo-Clavien classification

The medical records were thoroughly examined to register each deviation of the postoperative course. Every deviation from the standard treatment or the expected postoperative course was documented and regarded as a complication. Then, the management of the complication was recorded and used to grade according to the surgical Clavien-Dindo Classification (CDC) [2]. The CDC score classifies complications into five groups (Supplemental Table S1). The CDC grades the complication based on the severity of the required intervention. In case of several complications, CDC grading is based on the most extensive intervention.

### Statistics

All statistical analyses were performed using IBM SPSS Statistics 25 (Chicago, IL). Nominal and ordinal data are presented as absolute values and relative values in percentage. The results of the metric parameters are presented as means ± standard deviation (SD), median and range, if not otherwise indicated. The chi square test and nonparametric Mann–Whitney U test were used to compare the characteristics of two independent subgroups. Multivariate binary logistic regression models with stepwise entry were generated for the analysis of independent factors associated with increased risk for CDC complication (CDC ≥ I). Patients’ characteristics for the regression analyses were derived from those variables that were significant in preliminary univariate analyses (*p* < 0.05). In general, nominal p values of two-tailed tests are reported. P values < 0.05 were considered significant.

## Results

### Characteristics of the patients

The study sample was constituted from all 1465 surgeries performed in 2020. The majority of the patients were male (60.3%). The median age was 52 years. About one third were smokers (38.2%) and less than the half were drinking alcohol (44.7%). The majority had no relevant comorbidity (CCI0; 65.2%). The frequencies of CCI1, CCI2, and CCI3 were 13.0%, 13.6%, and 7.5%, respectively. The median body mass index (BMI) was 25.3. The patients stayed between 0 days (outpatient care) and 92 days (median: 3 days) in the hospital. More details and blood values are presented in Table 1.

**Table 1:** Patients’ characteristics

Parameter	*N*	%
All	1465	100
Gender		
Male	912	60.3
Female	553	37.7
Alcohol drinking	655	44.7
Smoking	559	38.2
ASA classification		
ASA I	371	25.3
ASA II	657	44.8
ASA III	210	16.9
ASA IV	3	0.2
ASA V	2	0.2
Unknown	222	15.2
Charlson Comorbidity		
0	955	65.2
1	190	13.0
2	199	13.6
3	110	7.5
5	1	0.1
6	5	0.3
7	3	0.2
Unknown	2	0.1
	**M ± SD**	**Median; range**
Age in years	46.45 ± 24.2	52; 98–0
Smoking in pack years	25.44 ± 20	20; 150–0.1
BMI	25.6 ± 6.6	25.3; 79.3–11.6
Outpatient/Inpatient duration in days	4.3 ± 4.5	3; 0–92
CRP in mg/l	9.9 ± 24.3	2.3; 314–0.6
Quick value in %	98.1 ± 14.1	100; 130–28
aPTT in sec	29.5 ± 8.4	28.9; 298–18.6
Thrombocytes pro µl	265 ± 79.2	255; 670–45
Creatinine in µmol/l	78.4 ± 32.2	73; 558–35
Glucose in mmol/l	6.2 ± 1.5	5.8; 19–3

M = mean; SD = standard deviation; BMI = body mass index

### Otorhinolaryngology and head and neck surgeries

An overview on the diseases and surgeries is given in Table 2. The diseases needing surgery were located most frequently in the oral cavity/pharynx (31.9%), the ear (15.0%), the nose (12.2%), and the larynx/thyroid (9.6%). The most frequent indications for surgery were: (suspicion of) a malignant tumor (38.0%), infection/inflammation (30.2%), benign tumor/mass (14.9%), or a sensory/functional disorder (13.1%). The most frequent types of surgery were: diagnostic endoscopy/biopsy (19.4%), ear surgery (14.8%), oral or pharyngeal surgery (12.1%), or nasal surgery (11.5%). The median time of surgery was 36 min (range: 3–509 min).

**Table 2:** Diseases and surgery

Parameter	*N*	%
All	1465	100
Localization of the disease		
Oral cavity/pharynx	468	31.9
Ear	220	15.0
Nose	178	12.2
Larynx/thyroid	140	9.6
Salivary gland	101	6.9
Paranasal sinus	93	6.3
Neck	71	4.8
Trachea/other airway	49	3.3
Eye	29	2.0
Other	116	7.9
Indication for surgery		
Malignant tumor/suspicion of malignant tumor	556	38.0
Infection/Inflammation	443	30.2
Benign tumor/mass	218	14.9
Sensory/functional disorder	192	13.1
Trauma	33	2.3
Cosmetic alteration	21	1.4
Other	2	0.1
Surgery*		
Diagnostic endoscopy/biopsy (1–6 to 1–8**)	284	19.4
Ear (5–18 to 5–20)	217	14.8
Oral cavity/Oropharynx/Hypopharynx (5–25 to 5–29)	177	12.1
Nose (5–21)	169	11.5
Neck (5–39, 5–40)	132	9.0
Paranasal sinus (5–22)	93	6.3
Salivary gland (5–26)	88	6.0
Nasopharynx (5–28)	68	4.6
Face/Skin (5–8, 5–9)	58	4.0
Larynx (5 − 3)	50	3.4
Trachea/Lung (5–31, 5–32)	46	3.1
Esophagus (5–42)	38	2.6
Eye (5–08, 5–16)	27	1.8
Thyroid (5–06)	13	0.9
Other	5	0.3
	**M ± SD**	**Median; range**
Duration of surgery in min	51.9 **±** 49.3	36; 3–509
Duration of follow-up in months	6.5 **±** 7.7	3; 0–30

*Primary surgery listed only, some patients received several procedures, **OPS-Codes in brackets;

### Assessment of the postoperative complications with the Clavien-Dindo classification

**Fig. 1:**
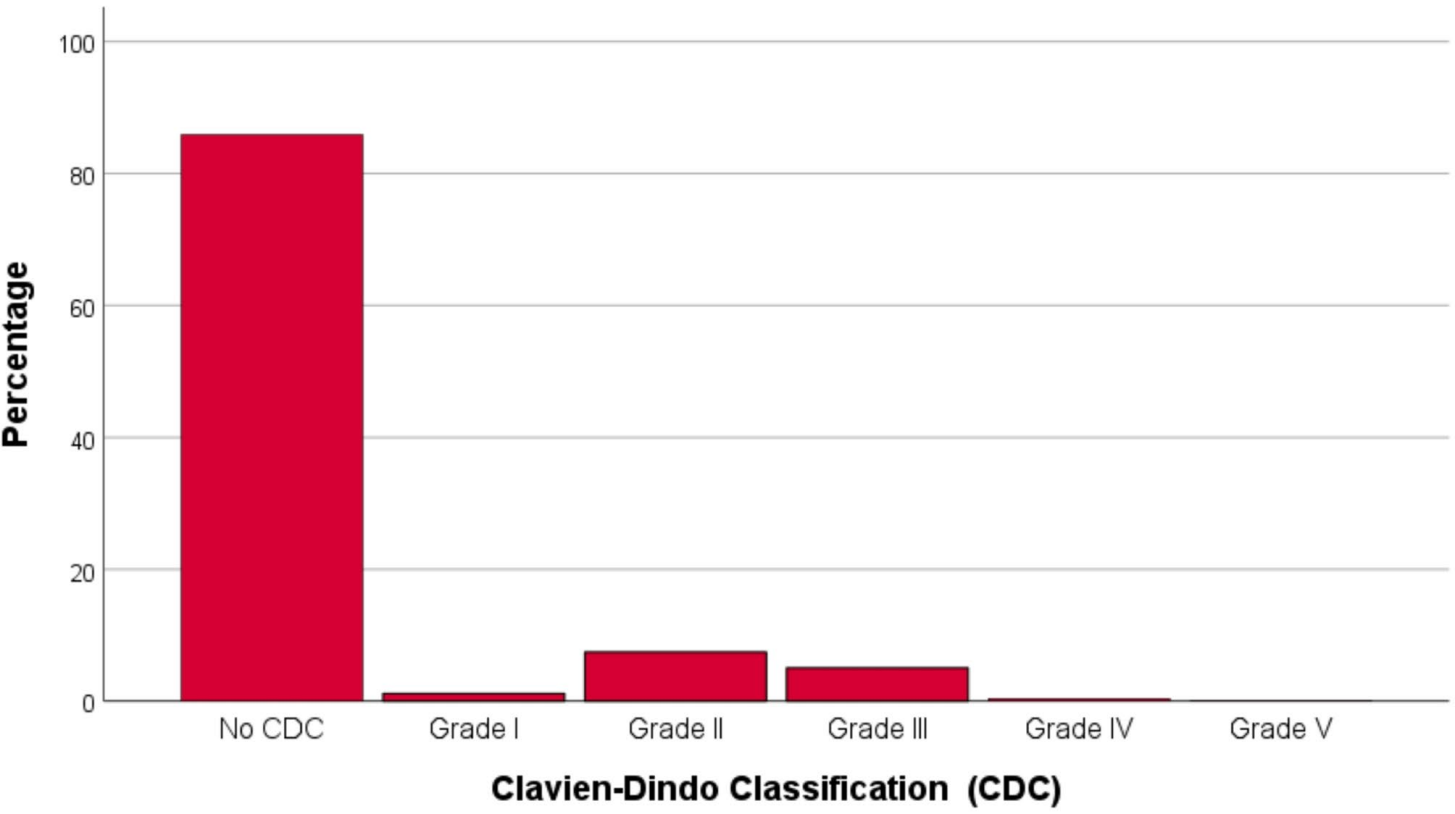
Distribution of the Clavien-Dindo classification (CDC) grades for the 1465 surgeries

**Fig. 2:**
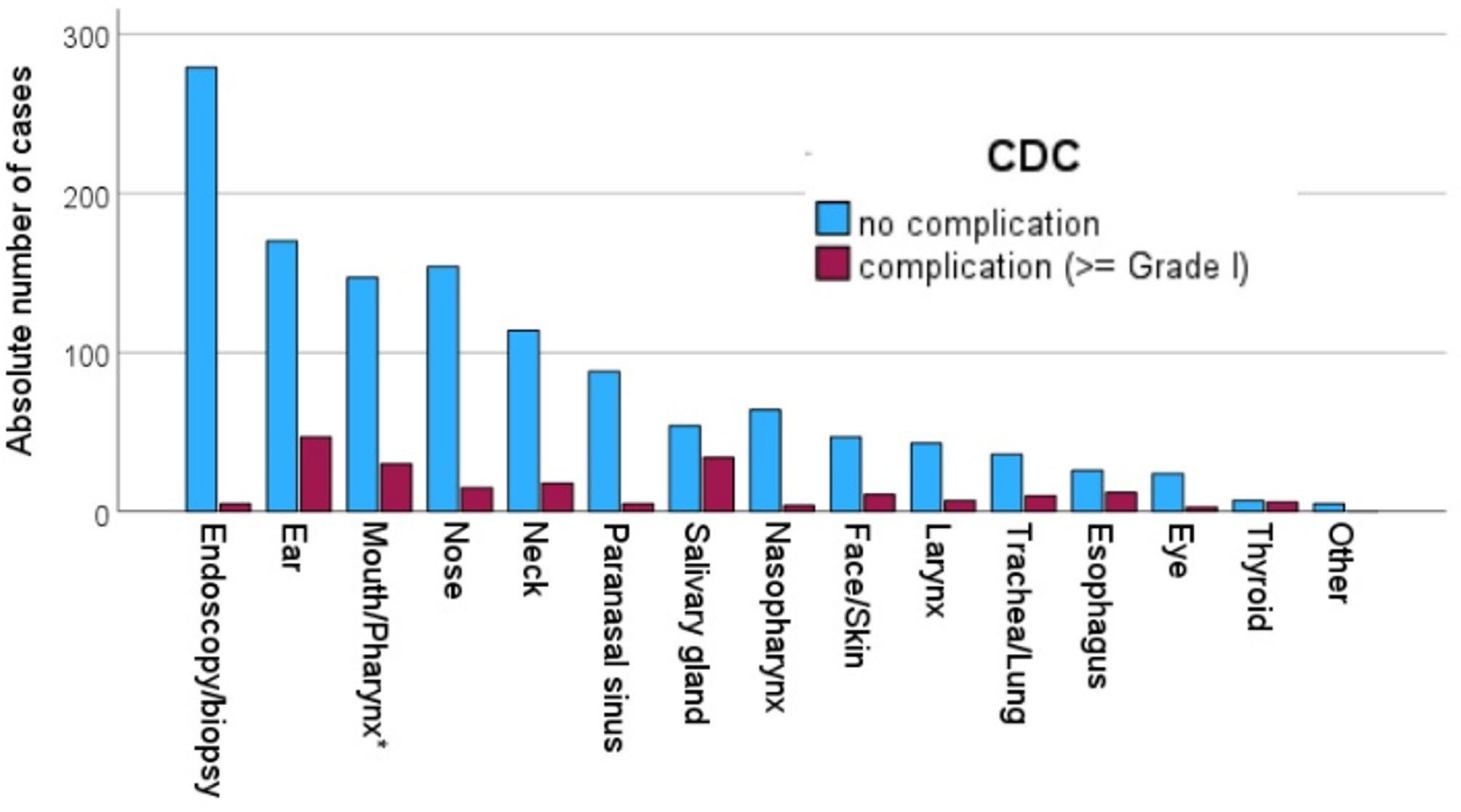
Number of cases with Clavien-Dindo classification (CDC) complications (≥ grade I) related to the surgery/surgery site. *Pharynx without nasopharynx

The severity of the postoperative complications is listed in Table 3. The vast majority of cases had no postoperative complication (85.9%; Fig. 1). The most frequent CDC classes were CDC grade II (6.9%) and CDC grade III (5.1%). CDC grade IV (0.4%) and CDC grade V (< 0.1%) occurred very seldom. The relative amount of CDC complications (≥ grade I) was highest in surgeries indicated for a benign tumor/mass (20.6%), followed by surgery for sensory/functional disorders (16.1%), and surgery for a (suspected) malignant tumor (11.7%). In relation to the operated organ, the relative amount of CDC complications (≥ grade I) was highest for thyroid surgery (46.2%), salivary gland surgery (38.6%), and esophageal surgery (31.6%; Fig. 2). The most frequent CDC criteria for the grading were extensive pharmacological treatment (9.7%) and surgical intervention (5.2%). A re-admission because of surgical complications was necessary in 3.7% of the cases.

**Table 3:** Clavien-Dindo classification (CDC) of postoperative complications

Parameter	*N*	%	
All	1465	100	
CDC			
No	1258	85.9	
Grade I	17	1.2	
Grade II	109	6.9	
Grade III	74	5.1	
Grade IIIa	3	< 0.1	
Grade IIIb	71	4.9	
Grade IV	6	0.4	
Grade IVa	6	0.4	
Grade IVb	0	0	
Grade V	1	< 0.1	
CDC criteria			
Minimal pharmacological therapy	50	3.4	
Physiotherapy	3	0.2	
Extensive pharmacological treatment	142	9.7	
Total parenteral nutrition	1	0.1	
Blood transfusion	8	0.5	
Surgical intervention	76	5.2	
Endoscopic intervention	7	0.5	
Radiological intervention	3	0.2	
Inhalation anesthesia	0	0	
Total intravenous anesthesia	0	0	
Intervention under general anesthesia	72	4.9	
Need of intensive care unit	6	0.4	
Single organ dysfunction	0	0	
Multi organ dysfunction	0	0	
Death	1	0.1	
Re-admission			
No	1027	70.1	
Yes	440	30.0	
Because of surgical complication	54	3.7	
Because of disease recurrence	92	6.3	
Other reason	294	20	

### Patients’ and surgical factors associated with postoperative complications

The univariate analyses of factors with association to postoperative complications assessed by CDC are shown in **Supplemental Table S2**. Smoking, the localization of the disease (salivary glands, ear), the indication for surgery, the type of surgery, and a longer duration of surgery were associated with a CDC ≥ I (all *p* < 0.05). From the pre-operative blood values, lower thrombocytes, higher creatinine, and higher glucose values were associated to CDC complications (all *p* < 0.001). Patients with CDC complications had a higher probability of re-admission (*p* < 0.001).

Three multivariate models (Tables 4 and 5) were calculated including: parameters concerning the patients’ characteristics (model 1), association to re-admission (model 2), and the indication for surgery and the type of surgical treatment (model 3). When analyzing the patient’s characteristics, only smoking remained as independent factor associated with higher probability of postoperative complications (Odds ratio [OR] = 1.526; confidence interval [CI] = 1.037 to 2.244; *p* = 0.032; Table 4). The risk of re-admission (model 2) was 2.9-fold higher for patients with postoperative complications (OR = 2.859; CI = 2.119 to 3.859; *p* < 0.001). Concerning the indication for surgery and the type of surgery (model 3; Table 5), and compared to a diagnostic endoscopy, the incidence of postoperative complications was higher for ear surgery (OR = 9.572; CI = 3.394 to 26.996; *p* < 0.001), esophageal surgery (highest risk: OR = 23.580; CI = 7.619 to 72.978: *p* < 0.001), external eye surgery (OR = 5.177; CI = 1.125 to 23.822; *p* = 0.035), surgery in the face/skin surgery (OR = 10.088; CI = 3.303 to 30.812; *p* < 0.001), laryngeal surgery (OR = 5.512; CI = 1.605 to18.930; *p* = 0.007), oral/pharyngeal surgery (OR = 6.888; CI = 2.444 to 19.410; *p* < 0.001), neck surgery (OR = 4.936; CI = 1.736 to 14.033; *p* = 0.003), salivary gland surgery (OR = 15.330; CI = 5.215 to 45.070; *p* < 0.001), thyroid surgery (second highest risk: OR = 21.060; CI = 4.838 to 91.665; *p* < 0.001), and trachea/lung surgery (OR = 11.248; CI = 3.404 to 37.173; *p* < 0.001). This risk was not higher after nasopharyngeal surgery (OR = 2.467; CI = 0.587 to 10.359; *p* = 0.218), paranasal sinus surgery (OR = 1.811; CI = 0.466 to 7.041; *p* = 0.391), and other surgery (HR = 0.000; CI = 0.000; *p* = 0.999).

**Table 4:** Independent associations between patients’ characteristics, treatment and the probability to have postoperative complications (CDC ≥ I)

Measure		OR*	95% CI* lower	95% CI* upper	*p*	
**Model 1: Patients’ characteristics**						
Smoking	No	1	Reference			
	Yes	1.526	1.037	2.244	**0.032**	
Localization disease	Eye	1	Reference			
	Ear	2.160	0.463	10.067	0.327	
	Nose	1.052	0.203	5.470	0.951	
	Paranasal sinus	0.455	0.075	2.765	0.393	
	Oral cavity/pharynx	1.018	0.216	4.798	0.982	
	Larynx/thyroid	0.616	0.120	3.174	0.562	
	Trachea/other airway	1.680	0.282	10.000	0.568	
	Salivary gland	4.537	0.952	21.637	0.058	
	Neck	0.476	0.071	3.214	0.447	
	Other	2.096	0.428	10.276	0.362	
Thrombocytes pro µl		0.999	0.996	1.001	0.335	
Creatinine in µmol/l		1.003	0.998	1.009	0.219	
Glucose in mmol/l		1.060	0.942	1.193	0.335	
**Model 2: Re-admission**						
Re-admission	No	1	Reference			
	Yes	2.859	2.119	3.859	**< 0.001**	

Multivariable binary logistic regression for the dichotomized outcome parameter CDC (no CDC complication versus CDC complication. Significant p-values (*p* < 0.05) in bold. OR = odds ratio; CI = confidence interval

**Table 5:** Independent associations between patients’ characteristics, treatment and the probability to have postoperative complications (CDC ≥ I)

Measure		OR*	95% CI* lower	95% CI* upper	*p*	
**Model 3: Indication and treatment**						
Indication surgery	Infection/Inflammation	1	Reference			
	Malignant tumor/ suspicion of malignant tumor	0.786	0.455	1.357	0.387	
	Benign tumor/mass	0.977	0.523	1.825	0.942	
	Trauma	0.895	0.182	4.414	0.892	
	Sensory/functional disorder	1.244	0.632	2.449	0.528	
	Cosmetic alteration	0.460	0.102	2.072	0.312	
	Other	1.031	0.000		1.000	
Surgery	Diagnostic endoscopy/biopsy (1–6 to 1–8*)	1	Reference			
	Ear (5–18 to 5–20)	9.572	3.394	26.996	**< 0.001**	
	Esophagus (5–42)	23.580	7.619	72.978	**< 0.001**	
	Eye (5–08, 5–16)	5.177	1.125	23.822	**0.035**	
	Face/Skin (5–8, 5–9)	10.088	3.303	30.812	**< 0.001**	
	Larynx (5 − 3)	5.512	1.605	18.930	**0.007**	
	Oral cavity/Oropharynx/ Hypopharynx (5–25 to 5–29)	6.888	2.444	19.410	**< 0.001**	
	Nasopharynx (5–28)	2.467	0.587	10.359	0.218	
	Neck (5–39, 5–40)	4.936	1.736	14.033	**0.003**	
	Nose (5–21)	2.704	0.826	8.858	0.100	
	Other	0.000	0.000		0.999	
	Paranasal sinus (5–22)	1.811	0.466	7.041	0.391	
	Salivary gland (5–26)	15.330	5.215	45.070	**< 0.001**	
	Thyroid (5–06)	21.060	4.838	91.665	**< 0.001**	
	Trachea/Lung (5–31, 5–32)	11.248	3.404	37.173	**< 0.001**	
Duration of surgery in min		1.009	1.006	1.012	**< 0.001**	

Multivariable binary logistic regression for the dichotomized outcome parameter CDC (no CDC complication versus CDC complication. Significant p-values (*p* < 0.05) in bold. OR = odds ratio; CI = confidence interval; *OPS-Codes in brackets

## Discussion

How common are complications occur after otorhinolaryngology or head and neck surgery? One might think that this question is easy to answer. The opposite is the case. Depending on the type and the site of surgery, the incidence of complications varied considerably [13]. Severity can vary from mild bleeding or minor wound infection in less than 5% of the cases to high-risk cancer salvage surgery with severe bleeding risk of up to 36% or a wound infection risk of up to 45% [13]. To solve this problem, the CDC does not classify the severity of the complication itself. CDC uses the efforts of the treatment to classify the complications. This allows for a comparison of different types of otorhinolaryngology or head and neck surgery within one cohort like in the present study, a comparison between patients from different departments of otorhinolaryngology, or even a comparison to other surgical disciplines.

Overall, 14.1% of the patients had complications CDC ≥ grade I in the presented unselected case series. Severe CDC complications (CDC ≥ grade III) were seen in 5.6% of the patients. Life-threatening complications (CDC ≥ grade IV) were rare with 0.5%. We are not aware of any other study analyzing an unselected series of otorhinolaryngology or head and neck surgeries. Monteiro et al. analyzed 371 patients undergoing head and neck surgery with microvascular reconstruction [4]. Overall, 59% of the patients had complications demonstrating the high probability of complication in a risk population. In a recent series from India, as much as 73% of 242 patients undergoing head and neck surgery (more details unfortunately not reported) had CDC complications. Concerning benign diseases, a CDC complication rate of 21% is reported for parotid gland surgery for benign tumors treated in Amsterdam [8]. The rate was higher in the present study with 38.6% for any kind of salivary gland surgery. The reason might be that both departments are centers for salivary gland surgery which leads to the allocation of difficult cases. The main reason for a CDC complication in the present study was a pharmacological intervention, specifically the application of a corticosteroids due to postoperative facial nerve palsy. No clear risk profile for CDC complications was found in the Dutch study population, except for an ASA score ≥ 2 for CDC ≥ 2 [8]. ASA was no relevant risk factor in the present study. For 402 children undergoing adenotonsillectomy a CDC complication rate (CDC ≥ 1) of 30.8% is reported [10]. Important risk factors were comorbidity, higher ASA score, and duration of surgery. Selecting only the children with adenotonsillectomy, we calculate also a CDC rate of 25%. This selection is too small in the present study to perform a multivariate analysis on risk factors on this subgroup. A CDC complication rate of 10.8% is reported after cochlear implant surgery [7]. This was higher with 30.3% in the present study. Finally, Smith et al. reported severe complication (CDC ≥ 3) for 21.6% of 185 patients undergoing vestibular schwannoma surgery [9]. The present series did not include such cases.

Smoking was the only patient characteristic that was independently associated with an increased risk of CDC complications. Smoking is a well-known risk factor for perioperative complications in head and neck surgery [14]. Patients were classified in the present study as smokers if they quit smoking ≤ 3 months before surgery. In some other studies, patients are not classified as smokers when they had smoked an average of less than 1 cigarette per day for less than 1 year [15]. Beyond otorhinolaryngology, head and neck surgery specific risk factors (like surgical site), the present study revealed surgery time as an important risk factor for CDC complications. This seems to be a general risk factor independent from the analyzed surgical discipline (for instance [16–18]),.

Due to the retrospective character of the present study, there were several limitations. ASA classification was missing for 15.2% of the patients. We cannot exclude that this has an influence on the results. To date, ASA class has not been a predictor of perioperative complications. Another limitation is that we do not know which surgeries were emergency surgery. One would expect the complication rate for emergency surgery to be higher than for elective surgery. The CDC grading was performed by one observer and controlled by a second observer. The CDC inter-observer reliability is higher for severe complications than for mild complications [4]. It would be advisable for future studies to involve more observers for the CDC classification. Furthermore, a patient cohort from 2020, i.e., a cohort treated within the COVID-19 pandemic, was analyzed. We did not have infection data from the patients. One retrospective trial suggested that at least COVID-19-positive head and neck cancer patients were at increased risk for severe perioperative complications [19]. It can only be stated that the unplanned re-admission rate in the present cohort was not different from the rate in 2015 in the same department [20]. An important aspect of surgical quality control is the monitoring of complications in surgical training. It must be taken into account that beginners tend to perform simpler surgeries and difficult procedures with a higher risk of complications are performed by experienced surgeons [21]. The CDC has so far been little used for this purpose. Recently, CDC was used as a quality measure while stablishing robotic bariatric surgery at an academic tertiary hospital [22].

The CDC (primarily designed for and by general and visceral surgeons) has limitations when used in otorhinolaryngology, head and neck surgery: Complications such as sensorineural hearing loss, loss of smell and facial nerve palsy are graded quite mildly as they usually do not require intensive care unit treatment [23]. However, these complications may be very severe for the patient. The CDC also does not include the reason for surgery. Most would probably agree, that a marginal mandibular nerve palsy after a parotidectomy for a small benign tumor is worse than the same complication in a patient undergoing a segmental mandibulectomy for advanced oral cancer [23]. Therefore, Morand et al. have recently developed an “indication to complication index” (ICI) including the discussed aspects [23]. Morand et al. first transformed the CDC into a numerical system and integrating loss of sensory organ into the system to better match with the spectrum of complications in otorhinolaryngology. Based on that, a new scale for surgical indications was developed and validated. For the new scale, surgical indications were divided in seven categories, grading lifestyle surgery (e.g., esthetic plastic surgery) as “1;” up to urgent surgery for a life-threatening condition (e.g., emergency tracheotomy for acute respiratory distress) received the highest grade (“7”). We now have to wait for further application of the new scale. Recently, Stone et al. published a novel Quality Improvement Classification System (QICS) [24]. QICS has 7 grades and combines the CDC with National Coordinating Council for Medication Error Reporting and Prevention (NCC-MERP) system. It has to be emphasized that the NCC.MERP does not classify complications but errors, reaching from errors not reaching the patient (no harm) over errors requiring an intervention (harm) up to errors contributing to the patient’s death. QICS is now integrating any deviation from normal treatment course, whether due to an error or a complication, and its degree of harmfulness in one classification. It remains to be seen whether the ICI or the QICS will find broader acceptance.

## Conclusions

The present study of 1465 cases gave an overview on surgical complications after a wide spectrum of otorhinolaryngology and head and neck surgeries using the Clavien-Dindo classification for grading of the severity of the complications. Important factors associated with postoperative complications were revealed. The data of the present study were a good starting point for further comparisons between hospitals, level of care settings, types of surgery, and of course, interventions to reduce postoperative complication after otorhinolaryngology - head and neck surgery.

## Electronic supplementary material

Below is the link to the electronic supplementary material.

Supplementary Material 1

## Data Availability

The original contributions presented in the study are included in the article and in the supplementary material. Further inquiries can be directed to the corresponding author.
