# Supplementary material for: Clavien-Dindo classification for assessment of complications after 1465 unselected otorhinolaryngology and head and neck surgeries in a university hospital: a retrospective observational study
Source: BMC Surg. 2025 May 29;25:235. doi: 10.1186/s12893-025-02970-1 (PMC12121247; doi:10.1186/s12893-025-02970-1)
Supplement: Supplementary file 1 — Supplementary Material 1 [file 12893_2025_2970_MOESM1_ESM.docx]

**Clavien-Dindo classification for assessment of complications after 1465 unselected otorhinolaryngology and head and neck surgeries in a university hospital: a retrospective observational study**

**Supplement Tables**

**Supplemental Table S1**

| **Supplemental Table S1.** Clavien-Dindo classification ^2^. | |
| --- | --- |
| **Grade** | **Definition** |
| Grade I | Any deviation from the normal postoperative course without the need for pharmacological treatment or surgical, endoscopic and radiological interventions.  Permitted therapeutic regimens include: drugs as antiemetics, antipyretics, analgetics, diuretics and electrolytes and physiotherapy. This grade also includes wound infections opened at the bedside. |
| Grade II | Pharmacological treatment required with other medications than the permitted complications of grade I; including blood transfusions and total parenteral nutrition. |
| Grade III | Requiring surgical, endoscopic or radiological intervention |
|  | Intervention not under general anesthesia |
|  | Intervention under general anesthesia |
| Grade IV | Life-threatening complication (including CNS complications) requiring ICU-management |
|  | Single organ dysfunction (including dialysis) |
|  | Multiorgan dysfunction |
| Grade V | Death of a patient |

**Supplemental Table S2**

| **Supplemental Table S2.** Univariate analysis of the association between patient’s and surgery characteristics and the occurrence of postoperative complication classified by the Clavien-Dindo classification (CDC). | | | |
| --- | --- | --- | --- |
| **Parameter** | **No CDC complication** | **CDC**  **complication (≥I)** | **p** |
| All | 1258 | 207 |  |
| Gender |  |  | 0.342 |
| Male | 777 | 135 |  |
| Female | 481 | 72 |  |
| Alcohol drinking |  |  | 0.115 |
| Yes | 552 | 103 |  |
| No | 706 | 104 |  |
| Smoking |  |  | **<0.001** |
| Yes | 457 | 102 |  |
| No | 801 | 105 |  |
| ASA classification |  |  |  |
| ASA I-II | 889 | 139 | 0.540 |
| ASA III-V | 180 | 35 |  |
| Unknown | 189 | 33 |  |
| Charlson Comorbidity |  |  | 0.549 |
| 0 | 828 | 127 |  |
| 1-2 | 329 | 60 |  |
| ≥3 | 99 | 20 |  |
|  | 2 | 0 |  |
| Localization of the disease |  |  | **<0.001** |
| Eye | 27 | 2 |  |
| Ear | 174 | 46 |  |
| Nose | 158 | 20 |  |
| Paranasal sinus | 88 | 5 |  |
| Oral cavity/pharynx | 419 | 49 |  |
| Larynx/thyroid | 129 | 11 |  |
| Trachea/other airway | 38 | 11 |  |
| Salivary gland | 65 | 36 |  |
| Neck | 64 | 7 |  |
| Other | 96 | 20 |  |
| Localization of the disease |  |  | **<0.001** |
| Salivary gland | 65 | 36 |  |
| Not salivary gland | 1193 | 171 |  |
| Localization of the disease |  |  | **0.002** |
| Ear | 174 | 46 |  |
| Not ear | 1084 | 161 |  |
| Indication for surgery |  |  | **0.036** |
| Infection/Inflammation | 381 | 62 |  |
| Malignant tumor/ suspicion of malignant tumor | 491 | 65 |  |
| Benign tumor/mass | 173 | 45 |  |
| Trauma | 31 | 2 |  |
| Sensory/functional disorder | 161 | 31 |  |
| Cosmetic alteration | 19 | 2 |  |
| Other | 2 | 0 |  |
| Surgery* |  |  | **<0.001** |
| Diagnostic endoscopy/biopsy (1-6 to 1-8) | 279 | 5 |  |
| Ear (5-18 to 5-20) | 170 | 47 |  |
| Esophagus (5-42) | 26 | 12 |  |
| Eye (5-08, 5-16) | 24 | 3 |  |
| Face/Skin (5-8, 5-9) | 47 | 11 |  |
| Larynx (5-3) | 43 | 7 |  |
| Mouth/Oropharynx/Hypopharynx (5-25 to 5-29) | 147 | 30 |  |
| Nasopharynx (5-28) | 64 | 4 |  |
| Neck (5-39, 5-40) | 114 | 18 |  |
| Nose (5-21) | 154 | 15 |  |
| Other | 5 | 0 |  |
| Paranasal sinus (5-22) | 88 | 5 |  |
| Salivary gland (5-26) | 54 | 34 |  |
| Thyroid (5-06) | 7 | 6 |  |
| Trachea/Lung (5-31, 5-32) | 36 | 10 |  |
| Re-admission |  |  | **<0.001** |
| Yes | 333 | 105 |  |
| No | 925 | 102 |  |
|  | **M ± SD** | **M ± SD** | **p** |
| Age in years | 48.2**±**23.8 | 51.2**±**22.3 | 0.100 |
| BMI | 25.6**±**6.5 | 24.8**±**5.7 | 0.188 |
| CRP in mg/l | 8.4**±**21.6 | 10.7**±**23.5 | 0.085 |
| Quick value in % | 98.7**±**13.1 | 98.3**±**14.8 | 0.006 |
| aPTT in sec | 29.6**±**10.3 | 29.5**±**3.5 | 0.876 |
| Thrombocytes pro µl | 268.9**±**72.9 | 260.27**±**87.0 | **<0.001** |
| Creatinine in µmol/l | 74.4**±**22.4 | 80.9**±**41.5 | **<0.001** |
| Glucose in mmol/l | 5.8**±**1.0 | 6.4**±**1.8 | **<0.001** |
| Duration of surgery in min | 46.8**±**42.3 | 82.9**±**72.2 | **<0.001** |
| Outpatient/Inpatient duration in days | 4.0**±**4.3 | 6.1**±**5.5 | **<0.001** |
